# Errata

**Published:** 2014-12-05

## Vol. 63, No. 46

In the report “Ebola Virus Disease Cluster in the United States — Dallas County, Texas, 2014” two errors occurred.

On page 1087, in the second paragraph, the first sentence should read, “On September 25, a man aged 45 years (patient 1), who had arrived in the United States from Liberia 5 days earlier, went to a Dallas County, Texas, emergency department with fever, initially 100.1°F **(37.8°C)** but increased to 102.9°F (39.4°C), abdominal pain, and headache (Figure).”

In the fourth paragraph, the first sentence should read, “**On October 10, a nurse (patient 2) previously involved in direct care of patient 1 had a sore throat; on October 11, patient 2 went to the emergency department with fever (100.6°F [38.1°C]) and** was confirmed to have Ebola by real-time PCR later that day.”

## Vol. 63, No. 44

In the report, “Arthritis Among Veterans — United States, 2011–2013,” several errors occurred.

On page 999, in the first paragraph, the fourth sentence should read as follows: “This report summarizes the results of these analyses, which found that one in **three** veterans reported that they had arthritis (**34.7%**) and that that **age-standardized** prevalence was higher among veterans than nonveterans across most sociodemographic categories, including sex (**age-standardized** prevalence among male and female veterans was 25.0% and 31.3%, respectively).”

Also on page 999, in the fourth paragraph, the first sentence should read as follows: “Veterans had a higher overall **crude** prevalence of reported arthritis than nonveterans, **34.7% (CI = 34.3–35.1)** versus **24.0 (CI = 23.8–24.1)**.”

On page 1000, in Table 1, the characteristics of race/ethnicity, highest educational attainment, employment status, annual household income, and body mass index should have been footnoted using the existing * footnote (i.e., “*Age-standardized to 2000 U.S. projected population [age groups 18–44, 45–64, and ≥65 years]; includes only those for whom age was reported.”). Also in Table 1, the n’s in the right sided column headings under “Overall” should read as “Overall (n = **1,420,290**),” Nonveterans (n = 1,277,596),” and “Veterans (n = **184,694**).”

Also on page 1000, in the first paragraph, the first two sentences should read as follows: “Among the 50 states and DC, the median state-specific **age-standardized** arthritis prevalence among veterans was 25.4% (range = **18.8% in Hawaii** to 32.7% in West Virginia) (Table 2, Figure). Among male veterans, the median **age-standardized** state-specific prevalence was 24.7% (range = 18.4% in Hawaii to 32.7% in West Virginia); among women the median was 30.3% (range = 22.4% in Hawaii to 42.7% in Oregon) (Table 2).”

Finally, in the summary box on page 1003, in the “What is added by this report?” section, the second sentence should read as follows: “The analysis found that **34.7%** of veterans reported having arthritis (**35.0%** among men and 31.3% among women) and that prevalence was higher among veterans than nonveterans across most sociodemographic categories.”

